# New products

**DOI:** 10.1155/S1463924687000403

**Published:** 1987


Journal of Automatic Chemistry/*Journal of Clinical Laboratory Automation*, Vol. 9, No. 4 (October–December 1987), pp. 199–205

### New products

### Bichromatic analysis

Technicon RA systems have three forms of bichromatic correction techniques: fixed, variable and special bichromatics. These can be used, under appropriate conditions, to estimate sample blank in place of a separate sample blank assay – thus saving time, sample, reagent and cuvettes.

Bichromatics involve the correction of the absorbance obtained at the primary wavelength by estimation of the interfering substances at a secondary wavelength where the analyte chromophore has no appreciable absorbance.

Using fixed bichromatics, the absorbance found at the secondary wavelength is multiplied by a bichromatic factor and subtracted from the primary absorbance. For example the Technicon hexokinase glucose method uses a primary wavelength of 340 nm, a secondary wavelength of 380 nm with a bichromatic factor of 1·200.

Variable bichromatic analysis is of use where a single bichromatic correction factor is not satisfactory for expected levels of non-reactive sample interferences, such as turbidity. The feature can also be used to reject a test result when non-reactive sample interferences exceed a preset level, as with the Technicon-defined chloride assay. This feature allows a second bichromatic factor to be applied if the absorbance at the secondary wavelength exceeds a preset limit.

Special bichromatic analysis, now available on the RA-XT system, is employed in cases where a linear variable bichromatic correction is not satisfactory, for example inorganic phosphorus.

Absorbance readings are taken at the secondary wavelength and scaled by appropriate constants before being applied to the primary absorbance. The algorithm employed uses constants K1 and K2, K1 being predetermined on clear analyte containing no interferent as the net absorbance at the secondary wavelength divided by the net absorbance at the primary wavelength. K2 is a bichromatic factor determined to give best agreement between a blank corrected value for the assay and the new procedure using the special bichromatic algorithm.

Using these features, either with Technicon-defined methods or user-defined methods, the operator can reduce the effects of interfering substances to a minimum, while at the same time minimizing operation costs.

*Further details from Francis Hooley, Technicon Instruments Company Ltd, Evans House, Hamilton Close, Houndmills, Basingstoke, Hampshire RG21 2YE, UK. Tel.: 0256 29181.*

### Liquid handling system

The liquid handling system launched by DBSS in September 1987 represents a new approach to automatic chemistry designed to increase accuracy, eliminate errors and save valuable professional time. Each of the automatic syringes, valves and peristaltic pumps is co-ordinated from a bench-top IBM PC or compatible microcomputer, and no independent means of control is required. DBSS has designed sTALK, its own control software package, which is fully compatible with BASIC and allows the user to define commands interactively. DBSS envisages the following applications for the system: automatic analysis, titration, crystallization, additive dosing and laboratory perfusion for fermentation or chromatography.

DBSS has already sold the system to several major clients in the industrial sector and to universities.

The DBSS Partnership, a three-man team, was set up in November 1986. It comprises two former members of Imperial College, Patrick Shaw Stewart, a biochemist, and Peter Baldock, an electronics expert, and also James Duguid—a designer from Loughborough University.

*Details from The DBSS Partnership, PO Box 1011, London W2 4PQ. Tel.: 01 727 3465.*

### Synchron

The Beckman Synchron™ CX™4 clinical analyser is a flexible clinical chemistry system, capable of analysing most general, protein and drug chemistries at a rate in excess of 215 tests/h in either stat, panel, profile, or batch mode. Users in hospitals, private and public clinics are provided with an operator interface in a choice of five languages which are German, French, Italian, Spanish and English. The CX4 incorporates the latest software logic thereby setting new standards in ease-of-operation.

The Synchron CX4 can hold up to 24 on-board chemistry, protein or drug tests, and can be interfaced to other Beckman clinical analysers such as the CX™3, to provide full electrolyte capability. Adding to the versatility of the system, the CX4 allows the creation and storage of up to 100 user-defined methodologies.

A menu of over 30 tests will be available – these are provided in barcode-labelled cartridges which control instrument set-up and simplify inventory management. Savings in terms of both time and reagent wastage are significant.

Samples for analysis are loaded continuously by an automatic 'Autoloader' which maximizes the throughput of the system. Stat samples can be introduced at any time.

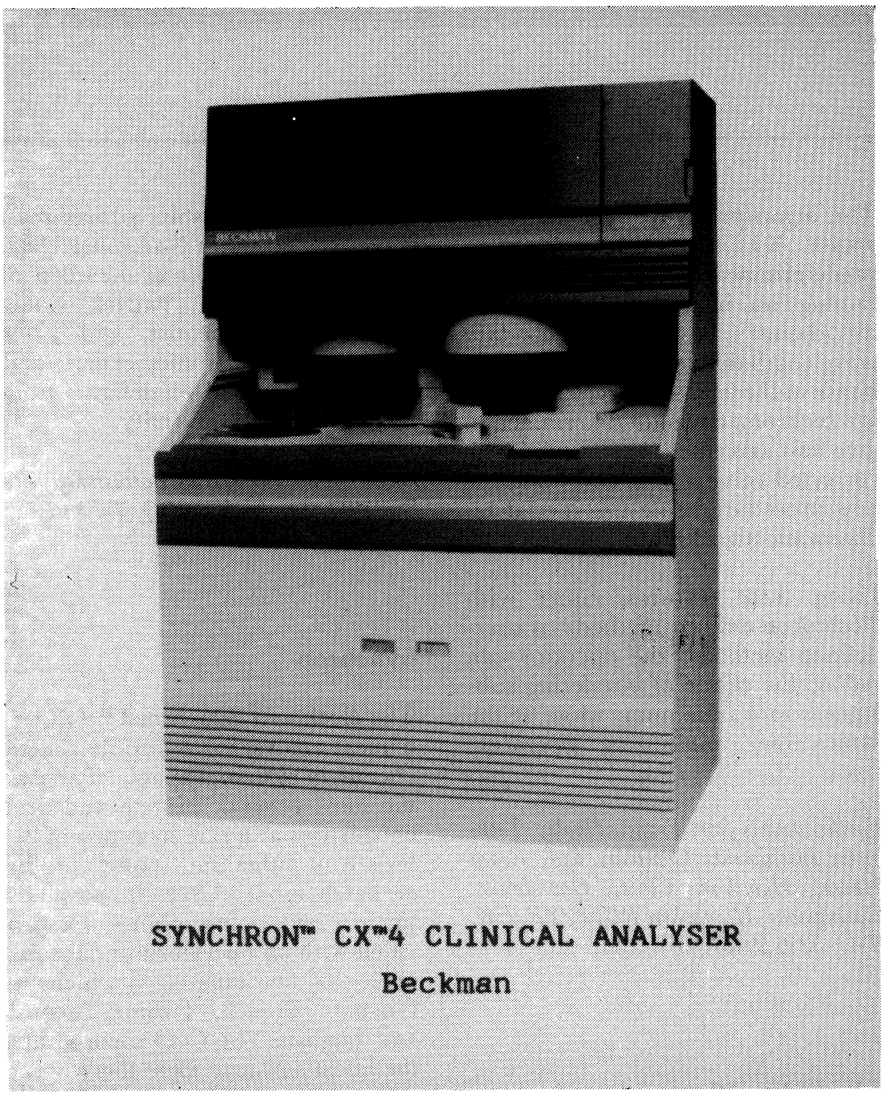

*Beckman's Synchron CX4 which features an operator interface that makes it simple to learn and easy to operate. Advanced software features, such as on-line QC, result formatting, and flexible patient demographics entry, mean that the Synchron CX4 is suitable for all testing environments. Moreover, extensive software-driven diagnostics and limited maintenance requirements reduce down-time to an absolute minimum.*

On-board refrigeration provides a 30-day reagent stability and the instrument's modular design provides easy access to customer serviceable areas.

Beckman's reagent technology provides accurate methodologies with long periods of calibration stability. In addition, the use of multi-analyte calibrators reduces the number of required on-board calibration materials.

*Details from Beckman Instruments, Inc., 2500 Harbor Boulevard, Fullerton, California 92634-3100, USA. Tel.: 714 871 4848.*

### Radiochromatography software

New Enterprises recently introduced a data handling system for the interpretation of chromatographic data in either analogue or digital form. The ISOCHROM is capable of operating with one or two radioactivity counting channels from an ISOFLO monitor, whilst a further two analogue channels are available for use with various detectors used in HPLC, FPLC and other flow monitoring techniques.

Specially written for operation with the IBM XT PC and compatibles, it provides a full range of functions required for comprehensive data handling and management including:

- On-line data collection in up to four channels
- Smoothing
- Automatic peak detection
- Automatic peak integration
- Zoom facility in both axes
- Automatic injector control
- Fraction collector control
- Synchronization of all four channels in time.

A foreground/background facility is also incorporated allowing a chromatogram to be run in the background whilst another program, such as 'Wordstar', is running in the foreground.

The system is therefore a powerful tool, enabling the user to present the final results in a form suitable for direct inclusion in reports.

*For further information contact Nuclear Enterprises Ltd, Bath Road, Beenham, Reading, Berkshire RG7 5PR, UK. Tel.: 0734 712121*

### Oximeter system

The new IL382 Oximeter System uses four wavelengths to look at all four haemoglobin forms and thus eliminates interferences other oximeters cannot measure. Instrumentation Laboratory's system uses the same proven optics and provides the same quality and performance as their Co-Oximeter system. With over 3000 operating worldwide, IL Co-Oximeter systems make up more than 90% of the world's total, and log an average of less than 1·5 service calls a year (better than 98·4% up-time).

However, the IL382 costs less than a Co-Oximeter because its designed for applications that require only a few key parameters, including total haemoglobin, oxygen saturation and oxygen content. It is ideal for operating-room anesthesiology, intensive

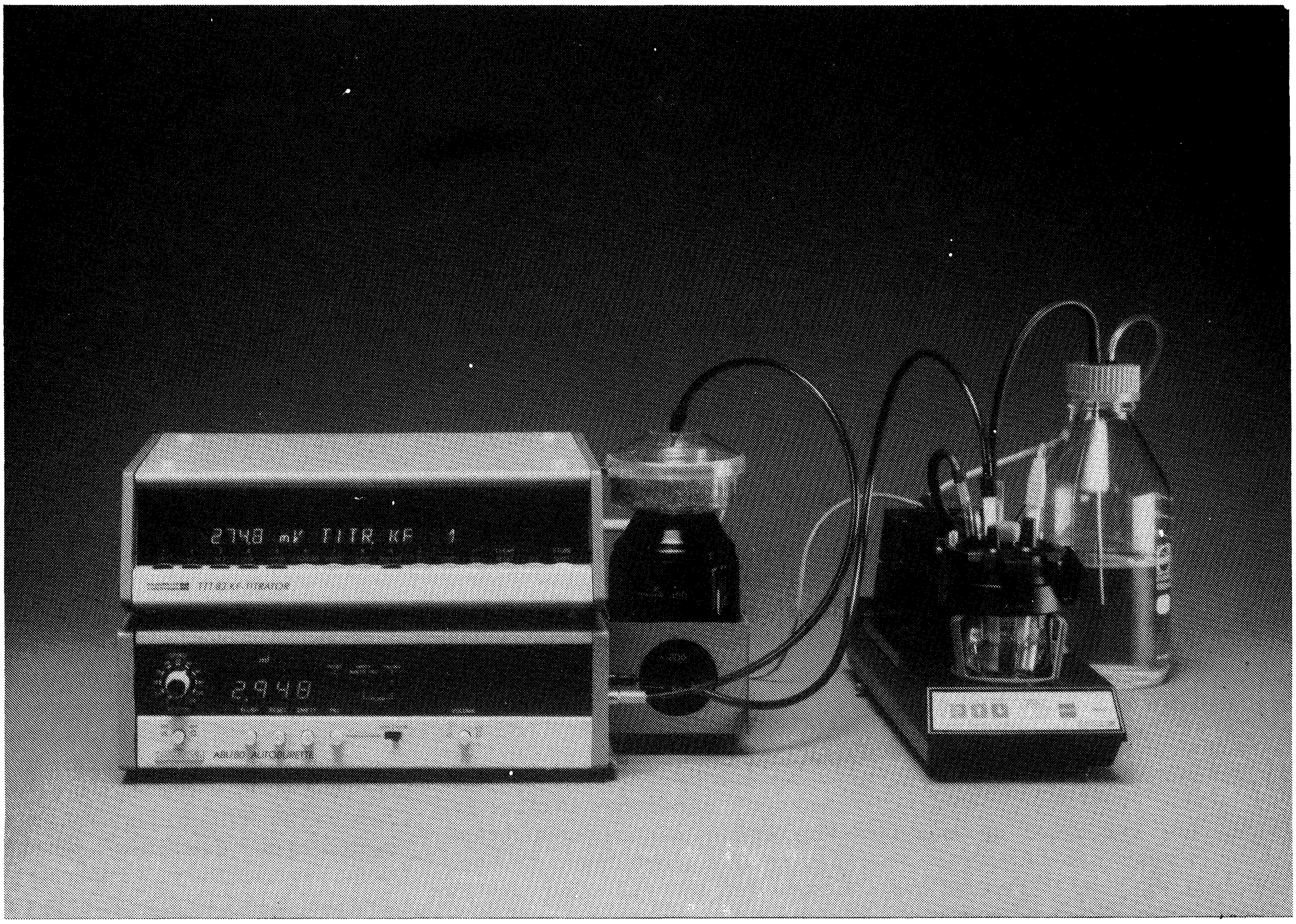

*The DTS830 Karl Fischer Titration System, which was launched in October 1987, has been designed specifically for water determinations with the aim of making operation as easy and as reliable as possible. The sample may be in any form and will still be easily introduced. The titration cell is tight and will withstand harsh chemicals. The cell is easily emptied using a built-in pump. Stirring is performed by a stirring magnet, but an optional propeller stirrer may be used. No movable part (but a magnetic field) is used for driving the magnetic bar. The DTS830 has a 20 character display with messages, parameters and results in user-selected units.*

*Four different user methods are available. For the user's convenience, special modes for titer determination and for stand-by titration have been incorporated. The latter enables the set-up to be in a ready state for a prolonged period. A built-in serial input/output enables on-line connection of balance, printer or computer. Effective burette volumes of 1, 2·5, 10 or 25 ml with a resolution down to 0·2 µl enables precise titration of samples with only some ppm of water. All types of Karl Fischer reagents may be used.*

*For further details from Radiometer Analytical A/S, Emdrupvej 72, DK 2400 Copenhagen NV, Denmark. Tel.: 1 69 63 11.*

care units, perinatology or cardiovascular laboratories.

The IL382 System also offers cost advantages over competitive oximeters. It doesn't require costly daily calibrations. And cost per test is only $0·09, compared with up to $0·50 per test for competitive instruments.

It requires only 85 µl of sample.

Sample/reagent path and the measuring cuvette are readily visible, allowing visual check on sample integrity. Comprehensive self-diagnostics alert the operator to faults by way of the alphanumeric display, allowing fast, easy troubleshooting.

*Detailed brochure is available free from Fischer Medical Division, Customer Inquiry Department, 711 Forbes Avenue, Pittsburgh, Pennsylvania 15219, USA.*

### HPLC fluorescence detector

A variable wavelength dual monochromator HPLC Fluorescence Detector from McPherson Instruments has the unique feature of E-Z interchangeable light sources and sampling modes. Increased sensitivity can be achieved with the Model FL-750/B by optimizing light source excitation energy in the region of spectral interest. Detection limits are down to 0·2 pg Anthracene. Analytical, preparative and microbore flow cells are available. In addition, the HPLC flow cell can be removed to accommodate a cuvette changer for spectral scans of multiple samples. Sampling accessories for TLC/GEL scanning and test-tube analysis are also offered.

*Details from McPherson Division of S.I. Corporation, 530 Main Street, Acton, Massachusetts 01720, USA. Tel.: 617 263 7733.*

### Digital refractometer

The DBX 55 covers the range 0–55% Brix and has a minimum scale of 0·1%. It has automatic temperature compensation from 5–40°C. The instrument will give a reading on a sample volume as little as 0·3 ml within 3 s. The refractometer is available with an optional printer which automatically prints the results for each sample, together with the temperature.

The DBX 55 should find wide application in the food and soft drinks industries. It can also be used for chemical and biochemical analysis and for quality control in the chemical, biochemical, pharmaceutical and cosmetic industries.

*Brochures from ChemLab Scientific Products Ltd, Construction House, Grenfell Avenue, Hornchurch, Essex RM12 4EH, UK. Tel.: 04024 76162.*

### Expert system

Logica has announced an expert system to support the formulation of industrial products. The system was developed from experience gained on the Product Formulation Expert System (PFES) project, which is part of the UK's Alvey programme.

The PFES Project, undertaken by a consortium of Shell Research Ltd, Schering Agrochemicals Ltd and Logica, investigated the application of knowledge-based techniques to the formulation of lubricating oils and agrochemicals.

Typical tasks which are undertaken in the formulation of these and other products, such as cleaning agents, paints, glues and foodstuffs, include selecting a suitable mix of component materials and testing, analysing and adjusting the mix until a saleable formulation is derived. This requires the scarce specialist skills of expert formulators.

A knowledge-based formulation system can aid experts' decision-making, rationalize their working practices, be used as a training aid by less experienced formulators and reduce the time spent on routine work. It also acts as a data management tool.

Logica developed the PFES demonstration around a formulation kernel, core software which can be used as a component when building customized systems.

*Details from Logica plc, 64 Newman Street, London W1A 4SE. Tel.: 01 637 9111.*

### High temperature oxygen analyser

The Servomex analyser, 1100H, has been designed for the analysis of clean, particulate free samples with dew-point temperatures up to 105°C. This high temperature measurement enables the 1100H analyser to determine the oxygen content of organic sample streams without the need for gas conditioning and, hence, eliminates errors due to condensation.

The analyser combines the advantages of microprocessor control electronics with the Servomex magnetodynamic transducer technology. This combination can be supplied in a range of configurations; the basic instrument is constructed as two units—

- A transducer unit, which is temperature controlled and houses the oxygen measuring cell.
- A control unit, which accepts the signal from the transducer unit and processes it to provide a digital output and a 0/4 to 20 mA current output.

This unit also provides power supplies required by the transducer, together with optional features such as alarm relay boards, data transmission and automatic calibration.

The two units can be separated so that the transducer unit is positioned close to the sample point to give good response time. If the separation exceeds 30 m then an interface unit is used to provide a digital link between the transducer and control units.

Comprehensive safety approvals from CENELEC and BASEEFA enable the use of the 1100H analyser in Zone 1 hazardous areas and with flammable gases. The Transducer Unit is certified EEx d ia IIC T3.

Standard features include battery protection of memory to guard against mains-power failure; choice of seven spans and zero offset to give several hundred output ranges; digital display with wide dynamic range, 0–100% oxygen and 0·1% resolution and a response time of less than 5 s.

A range of constructional materials may be used for the sampling system to give optimum chemical resistance to the constituents of the sample gas.

*Further information from Neil Blackford, Servomex Ltd, Crowborough, Sussex TN6 3DU, UK. Tel.: 08926 2181.*

### Filters to protect instruments

Filters to protect analysers from contaminated liquid or gas samples, or to purify air and gas used to operate instruments and actuators, are described in detail in new literature from Balston Ltd of Maidstone. A series of brochures give descriptions, illustrations, specifications, applications data and actual case histories covering a number of industries.

In applications, such as vehicle emmission analysers and on-line analysers, contaminated samples are often the most frequent cause of problems. Selection of the correct filter can minimize downtime by assuring complete removal of liquids and solids from gas samples, or gas bubbles from liquid samples.

Instrument clogging, corrosion and failure can be greatly reduced by removal of all oil, water and dirt from the air or gas used for operation. Many Balston filters are of the coalescing type, which means that the liquids removed are drained away continuously. This results in a long useful life, operating at full efficiency. Air purified by Balston filters has been proven pure enough for spinning and cooling samples of extremely sensitive NMR analysers where trace contaminants cannot be tolerated.

*More information from Balston Ltd, Monckton's Lane, Maidstone, Kent, UK. Tel.: 0622 52201.*

### DCP

ARL have updated the Spectraspan. The performance of this instrument for elemental analysis is proven; the rugged Direct Current Plasma Source is inexpensive to operate and easily handles all samples and matrices; the ultra high resolution Echelle spectrometer is the heart of a bench-top system which provides multi-element, optimum sensitivity measurements. The new instrument, the Spectraspan VB, now controls the measurement and processes the data with an IBM-PC-XT. Menu-driven software programs and large storage capacity allow application-specific methods development – from measurement to print-out.

The 20 element cassettes are easily interchangeable for different sample types and a sequential cassette gives the flexibility for unexpected samples. Qualitative and semi-quantitative analyses are made possible with a spectrographic accessory.

*Details from ARL Applied Research Laboratories SA, En Vallaire, 1024 Ecublens, Switzerland. Tel.: 021 34 97 01.*

### Direct injection nebulizer from P S Analytical in Europe

For most elemental analysis systems, such as ICP, DCP and atomic absorption, the weakest link is the sample introduction mechanism. The new Cetac Din 200 Direct Injection Nebulizer is a viable alternative to conventional pneumatic nebulizers. In addition, it provides a logical interface for flow injection analysis systems and HPLC applications. Because of its small dead volume and high mass transport the normal nebulizer limitations are removed. The Cetac Din 200 has been developed from research at Iowa State University, USA. With such a high mass transfer, it is possible to analyse samples with as little as 20 μl of sample with precision in the range of 2–3%. The table below lists the limits of detection by both FIA and continuous flow ICP for the Din 200 and a conventional pneumatic nebulizer.

The Din 200 is available throughout Europe from P S Analytical Ltd as a simple nebulizer, or as a complete system encorporating the nebulizer, HPLC splitter, injection valve, pump and heater.

*For further information contact: P S Analytical Ltd, Arthur House, Far North Building, Cray Avenue, Orpington, Kent BR5 3TR, UK. Tel.: 0689 31632 Telex: 265502 PSA G*

### Sadtler retention index library

Following a licensing agreement between Hewlett-Packard and Sadtler Research Laboratories of Philadelphia, the Sadtler standard capillary GC retention index library is now available as a data-base on the HP analytical GC workstation. Over 2000 chemical compounds are included in the library, providing a comprehensive and authoritative data-base for GC users.

Chromatographic data obtained on the HP 5895A gas chromatograph can be manipulated, stored and retrieved on the workstation, an HP 9000 Series 300 scientific computer. Among the information obtained may be retention index data, calculated from the specific peaks for which identification is requested by the user. The Sadtler data-base is searched for matches, which are listed in order of match quality based on the normal distribution.

The data-base may also be searched for entries with a specified name, molecular formula or retention index.

*More information from Tina Mears, Analytical Instrument Group, Hewlett-Packard Ltd, Miller House, The Ring, Bracknell, Berkshire RG12 1XN, UK.*

### CRP

C-Reactive Protein (CRP), an acute phase protein normally present in serum at low concentrations, becomes elevated in response to many inflammatory situations, tissue injury such as myocardial infarction, and viral and malignant neoplasia. Although elevated serum CRP levels are a non-specific response, serial measurements of CRP levels can be a valuable aid to diagnosis, differential diagnosis and management of the specific condition causing the elevation.

Technicon's RA system's CRP method is based on the reaction between an antibody and sample CRP. Human CRP and specific antibody react in dilute solution forming

*Estimated limits of detection by both FIA and continuous flow ICP for the DIN 200 and pneumatic nebulizer.*

| Element | Analytical wavelength (nm) | Relative limits of detection (ng/ml) Direct injection FIA | Direct injection Continuous | Pneumatic FIA | Pneumatic Continuous |
|---|---|---|---|---|---|
| As | 193·7 | 41 | 40 | 91 | 80 |
| Se | 196·0 | 37 | 52 | 94 | 81 |
| Hg | 194·2 | 20 | 22 | 27 | 16 |
| Pb | 220·4 | 28 | 28 | 31 | 27 |
| Cr | 205·6 | 12 | 12 | 13 | 12 |
| Zn | 213·9 | 2 | 2 | 4 | 4 |
| Fe | 238·2 | 1 | 4 | 3 | 3 |
| Cd | 214·4 | 4 | 1 | 5 | 4 |
| Mn | 257·6 | 1 | 1 | 1 | 1 |
| Ba | 445·4 | 1 | 2 | 3 | 2 |

an insoluble complex which is quantitated turbidimetrically at 340 nm. Polyethylene glycol is used to accelerate the formation of the antigen-antibody complexes.

The method provides rapid, precise and accurate test results with optical interferences from lipaemic, icteric or haemolysed samples being minimized by the final dilution of the sample and by internal blank correction.

The kit available now from Technicon is suitable for 140 tests with working reagents being prepared as required. Stock reagents are stable until the expiration date printed on the product label and working reagents for two weeks. SETpoint CRP calibrator is also available and each set will provide calibration for 40 weeks when calibrated once every two weeks.

The provision of this test widens the range of specific protein assays available to 13 with further expansion planned in the near future.

*Details from Francis Hooley at Technicon, see p. 199.*

### A graphics spreadsheet for signal analysis

Version 1·03 of the DADiSP Worksheet, the first technical spreadsheet software for digital signal processing, is now available in the UK from Adept Scientific of Letchworth, Hertfordshire. DADiSP (Data Acquisition and Digital Signal Processing) offers over 150 functions for displaying and analysing waveforms, calculating and presenting data with the power and flexibility of a spreadsheet. Complex signal processing chains are created in windows, allowing full worksheets to be built up and stored quickly and easily. The new enhanced version adds the facility of running external programs within the DADiSP environment.

The DADiSP Worksheet is designed to process and manipulate any type of digital signal displayed as a waveform, and therefore has applications in chemistry, mechanical testing, materials testing, electronics, physiology and many other disciplines. It does for scientists and engineers what spreadsheets do for businessmen, eliminating time-consuming recalculation, reconfiguration and waveform analyses. Complex signal processing is easy to understand, with different signals and the mathematical relationships between them displayed graphically in windows.

The menu-driven DADiSP Worksheet allows signals to be cursored, zoomed, expanded and compressed, and loaded into windows. Full background data on any signal can be displayed at the touch of a key; and both raw and derived signals presented in graphical or tabular form. DADiSP offers a broad range of functions for data transformation, including FFTs, waveform generation, statistical analyses, signal calculus, peak analysis and signal editing. New data may be loaded into windows, and the worksheet is then immediately updated to incorporate the changes, with each window recalculated to give true 'graphic spreadsheet' flexibility. Like spreadsheets, DADiSP Worksheets may be saved to and reloaded from disk.

Related signals and formulae are grouped into convenient Labbooks. Individual users of the DADiSP Worksheet can thus keep their work

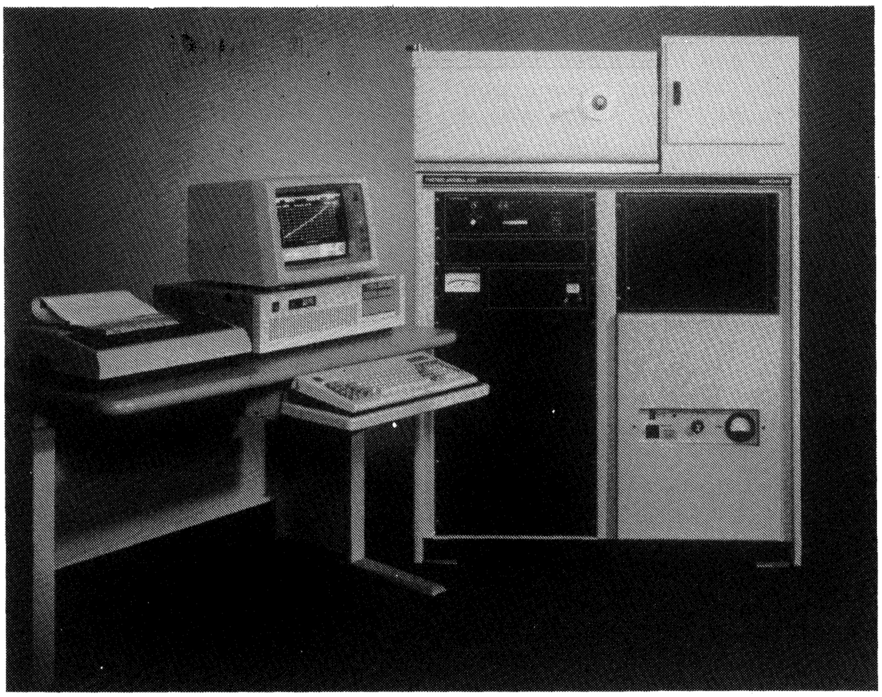

*The AtomComp 81 Direct Reading Spectrometer is capable of simultaneously determining up to 61 elements using an IBM PC/AT host computer as a user interface device. The operator interacts with the computer by using 10 pre-programmed soft keys and a screen format with 'windows' for entry of information. Comprehensive help screens guide the user through the set-up and execution of an analytical program. A learn mode is available to execute multiple functions with a single keystroke.*

*Spectral scans can be displayed on the enhanced resolution colour CRT. Analytical data is stored in an integrated environment which includes a data-base, spreadsheet, and word processor for generation of custom reports.*

*The Excitation Source for the AtomComp 81 can be either an Electronically Controlled Waveform Source (ECWS) spark, or a DC arc, or both in a combination system. The AtomComp 81 can also be equipped with a combination of the ECWS and Inductively Coupled Plasma. The ECWS vaporizes the metal sample and generates a metal aerosol. The ICP source then excites the aerosol to emission for a solid sampling plasma system.*

*For additional details contact Thermo Jarrell Ash, 590 Lincoln Street, Waltham, Massachusetts 02254, USA.*

separate from their colleagues', and call up specific Labbooks as required. User-defined functions may be generated through DADiSP's macro definition facility, to completely automate DADiSP operation.

DADiSP Version 1·03 features the DSP Pipeline, which allows external programs to be run alongside DADiSP. Data acquired through a program such as Labtech Notebook may be imported directly into a DADiSP analysis worksheet; and similarly, data from a DADiSP window may be exported to an external set of analysis or filtering algorithms and re-imported after modification. Third party IEEE 488, RS232 and plotter drivers may also be run from DADiSP using the DSP Pipeline.

The DADiSP Worksheet is available to run on any IBM PC or compatible with 640 K of RAM and two disc drives. It is fully compatible with either CGA/EGA or Hercules graphics.

*Product enquiries to Elaine Bragg, Adept Scientific, Letchworth Business Centre, Avenue One, Letchworth, Hertfordshire SG6 2HB, UK. Tel.: 0462 683355.*

### PS Analytical launch new automatic mercury analyser

At Lab Week 87, held in October, PS Analytical demonstrated the first in a line of complete instruments. Designed to measure the level of mercury at the sub ppb level, the system comprises the PSA 10·003 hydride generator linked to a new PSA 10·022 fluorescence monitor. The latter has been developed in conjunction with Spex Industries. The instrument is automatically fed from the PSA 20·020 large volume autosampler. Detection levels in the region of 0·05 ppb are readily obtainable. The mercury vapour is generated by the PSA 10·003 and transferred into the fluorescence monitor via a unique interface. The flow of mercury vapour is also shrouded in a flow of gas to prevent any quenching of the fluorescence signal. The system can also be easily adapted for use as a process stream analyser with level monitoring and alarm settings.

PS Analytical also exhibited its complete range of ICP, AA and DCP accessories, as well as the latest developments in DC plasma spectroscopy. Specifically, it showed the versatility of the large volume Autosampler and Valve Switching Data Transfer Interface, as building blocks for Laboratory Automation systems for individual customer requirements.

*For further information contact P. B. Stockwell, PS Analytical Ltd, Arthur House, Far North Building, Cray Avenue, Orpington, Kent BR5 3TR, UK. Tel.: 0689 31632.*

### Random access analyser for Emit drugs of abuse assays

Syva UK have announced a laboratory instrument for the detection of drugs of abuse in urine. The ETS System is a completely integrated analyser, which consists of a photometer unit, fluid handling system and built-in computer. The instrument is designed specifically for Emit dau Assays, which are used for analysing urine samples for commonly abused drugs. Assays for the following drugs are now available:

Amphetamines
Barbiturates
Benzodiazepines
Cannabinoids
Cocaine metabolites
Methadone
Methaqualone
Opiates
Propoxyphene
Phencyclidine (PCP)

The Ets analyser is capable of processing up to 16 urine samples in a single run. Either up to six assays may be tested on each urine sample, or the random access mode can be used to select assays individually.

Once samples and assay reagents are loaded on the instrument, all procedural steps are carried out automatically.

Results are printed out at a rate of 60 tests per hour. Output can be directed to the internal printer, or to an external computer for storage and re-formatting, if required.

*For further information contact Syva UK, Syntex House, St Ives Road, Maidenhead, Berkshire SL6 1RD, UK. Tel.: 0628 70969.*

### Multi-element AAS from Perkin-Elmer

The Model 2100 atomic absorption spectrometer is the first fully IBM PC controlled instrument on the market.

High performance is achieved using a unique optical design which combines the stability of double beam instruments with the energy throughput of single beam systems.

The Model 2100 can determine up to 18 elements in 50 samples with fast throughput which will ensure conveniences and cost savings for 2100 users.

Automatic lamp turret, burner positioning, gas box and flame sensing are of course standard features.

Using Perkin-Elmer's unique 'quick change mount' the user can change from flame to furnace operation in less than 3 minutes. Good background correction is vital in many applications and Perkin-Elmer's unique bracketing method of correction is used to ensure accuracy and precision even at very high absorbances.

The IBM or Epson PC controller allows the user to set and adjust any parameter via the keyboard. Once a method is developed it can be stored on floppy disk to be recalled at a later date. Stored data can be reformatted to the user's special requirements using either Perkin-Elmer's own Data Management Software or commercially available programmes such as Lotus 1, 2, 3 etc., and printed out as a completed report.

To complement the introduction of the new AA, Perkin-Elmer have introduced a furnace and autosampler. The Model HGA700 is designed to be interfaced and autosampler. The Model HGA700 is designed to be interfaced directly to either the Model 2100 or the Model 1100B. Rapid graphite furnace signals are fully resolved on the High Resolution Display screen of the Epson PC or IBM PC controller. Background corrected analyte signals (solid line) and background-only signals (dashed line) are shown simultaneously to provide complete analytical information.

Analyte and background signals can be scaled individually before or after a run. The software allows for simplified methods development and parameter optimization.

The AS70 is the furnace autosampler; features include:

- Simplified programming from the spectrometer keyboard.
- Automatic set-up of all autosampler parameters from stored methods.
- Automatic claibration for convenience and time saving.
- Improved calibration accuracy with multiple standards.
- Variable pipetting speeds for handling viscous samples.

*For further information please contact Perkin-Elmer Ltd, Post Office Lane, Beaconsfield, Buckinghamshire HP9 1QA, UK.*

### British companies score success with Rhine pollution

Three British companies in collaboration with a German scientific instrument factor, have succeeded in obtaining the contract for a major water analysis system for the Weisbaden-Hessische Landesanstalt Fuer Umwelt-Laboratory in West Germany. Headed by Dr Papke, the Weisbaden Laboratory monitors the quality of the Rhine water and all surface waters in the county of Hessen (FR Germany). The new analytical system will perform over 50 000 determinations per month at a rate of nearly 1000 per hour.

The contract was awarded to S.E.S. GmbH, who are using ChemLab Instruments System 4 multi-channel analyser, at the heart of the equipment. The 12 channel analyser is fed by a high volume automatic sampler designed by P. S. Analytical, and made by ChemLab Manufacturing Ltd. Data handling is by the Trivector dataflow system which was developed in conjunction with ChemLab Instruments. S.E.S. have added sophisticated software, together with specialist sample handling techniques, to produce a fully automated system.

### DNA synthesis

New literature published in October by Beckman describes instrumentation, reagents and software available for DNA synthesis. Issued as a series of bulletins, the literature provides details of the latest instrumentation —the System 200A and the System 100 Series.

The automated 200A offers the utmost user flexibility at the lowest cost per cycle and is ideally suited to the work of researchers not wishing to make a large investment for an extensive system, yet have a significant need to synthesize DNA in quantities which would make manual methods uneconomical. With the 200A up to four different DNA fragments can be synthesized 24 hours a day.

The new 100 Series is a manual instrument capable of high performance with up to four columns and is most suitable for researchers requiring a 'personal' synthesis or only an occasional sequence as well as for teaching the chemistry of DNA synthesis. The instrument also serves as an efficient 'back-up' system and where the use of an alternative chemistry or alien monomers is needed. It will also be welcomed by those needing occasional oligonucleotides in large amounts for physical studies.

These instruments are fully supported by the Beckman range of high quality reagents and chemicals and the company's service and applications back-up.

*More information from Beckman Ltd, Progress Road, Sands Industrial Estate, High Wycombe, Buckinghamshire, UK. Tel.: 0494 41181.*

### ARC/SPARK emission spectrometry

A 20-page colour brochure for the AtomComp 81™ ARC/SPARK Direct Reading Spectrometer is now available from Thermo Jarrell Ash. The AtomComp 81 Emission Spectrometer is capable of simultaneously determining up to 61 elements in both ferrous and nonferrous materials. The system features ThermoSPEC software and an IBM Personal system/2 model 50 microcomputer. The AtomComp 81 provides complete analytical results in less than 1 min, allowing close control of expensive charge and alloying materials.

ThermoSPEC software is easy-to-use, providing simplified methods set-up and calibration, automatic curve fitting, QC and limit checking. It also includes a built-in report generator, and ENABLE™, an integrated word processing, spread sheet, data base management, and a telecommunications package.

*For additional details, phone 617 520 1880 or write to Thermo Jarrell Ash, 8E Forge Parkway, Franklin, MA 02038, USA.*

### Filling process control

With the new METTLER Stat Pac-M, random samples can be checked right next to the filling machine, and at the same time the weight readings can be statistically evaluated and the results printed out. The system also continuously logs the results of the random checks, so providing a record of the totals. The read-out appears on the display of the balance.

StatPac-M has a choice of seven tolerance systems, which greatly simplifies the checking of packaged products. The system also calculates tolerances required by law, for example EEC fill quantity regulations.

When filling liquids, StatPac-M includes density in calculation. If required, the tolerances can be defined at will, and even asymmetrically. This also applies to the size of the sample. All definitions are safely stored against power loss.

*Details from Mettler Instrumente AG, CH 8606 Greifensee, Switzerland*

